# A mixed methods assessment of knowledge, attitudes and practices related to aflatoxin contamination and exposure among caregivers of children under 5 years in western Kenya

**DOI:** 10.1017/S1368980023000150

**Published:** 2023-12

**Authors:** Abigael O Awuor, Gati Wambura, Isaac Ngere, Elizabeth Hunsperger, Clayton Onyango, Godfrey Bigogo, Lauren S Blum, Peninah Munyua, M Kariuki Njenga, Marc-Alain Widdowson

**Affiliations:** 1 Division of Global Health Protection, US Centers for Disease Control and Prevention, Nairobi, Kenya; 2 Washington State University Global Health Program, Nairobi, Kenya; 3 Kenya Medical Research Institute, Centre for Global Health Research, Kisumu, Kenya; 4 Paul G Allen School of Global Animal Health, Washington State University, Pullman 99164, USA; 5 Institute of Tropical Medicine, Antwerp, Belgium

**Keywords:** Aflatoxin contamination, Post-harvest agricultural techniques, Knowledge, Attitudes and practices, Quantitative and qualitative methods, East Africa

## Abstract

**Objective:**

Identifying factors that may influence aflatoxin exposure in children under 5 years of age living in farming households in western Kenya.

**Design:**

We used a mixed methods design. The quantitative component entailed serial cross-sectional interviews in 250 farming households to examine crop processing and conservation practices, household food storage and consumption and local understandings of aflatoxins. Qualitative data collection included focus group discussions (*N* 7) and key informant interviews (*N* 13) to explore explanations of harvesting and post-harvesting techniques and perceptions of crop spoilage.

**Setting:**

The study was carried out in Asembo, a rural community where high rates of child stunting exist.

**Participants:**

A total of 250 female primary caregivers of children under 5 years of age and thirteen experts in farming and food management participated.

**Results:**

Study results showed that from a young age, children routinely ate maize-based dishes. Economic constraints and changing environmental patterns guided the application of sub-optimal crop practices involving early harvest, poor drying, mixing spoiled with good cereals and storing cereals in polypropylene bags in confined quarters occupied by humans and livestock and raising risks of aflatoxin contamination. Most (80 %) smallholder farmers were unaware of aflatoxins and their harmful economic and health consequences.

**Conclusions:**

Young children living in subsistence farming households may be at risk of exposure to aflatoxins and consequent ill health and stunting. Sustained efforts to increase awareness of the risks of aflatoxins and control measures among subsistence farmers could help to mitigate practices that raise exposure.

Aflatoxins produced by strains of *Aspergillus flavus* and *Aspergillus parasiticus* are common contaminants of grains and other crops in tropical and subtropical regions and have been shown to have a substantial impact on food production, food security and nutritional value^(1–4)^. Chronic dietary aflatoxin exposure can lead to hepatic carcinoma, immune system suppression or immunosuppression and child stunting^(4–6)^ while ingestion of highly contaminated grains can cause outbreaks of acute liver failure and death^(2,3,7)^. Widespread exposure in Africa and Asia has led the WHO to recognise aflatoxins as a global food safety concern, especially among rural subsistence farmers^(7,8)^.

In Africa, certain climatic conditions and agricultural practices (i.e. poor harvesting, food processing and storage practices) favour conditions for fungal contamination of cereals, especially maize when stored with high moisture content^(1,3,4,9)^. Research has shown that extremely toxigenic fungal communities exist in several east African countries, including Kenya^(10)^. Historically, Kenya has been one of the worst affected countries by aflatoxicosis outbreaks, particularly eastern Kenya^(9,11)^. Multiple studies in Kenya have reported the presence of high concentrations of aflatoxins in homegrown cereals and animal milk samples with the potential for chronic and acute exposure in humans^(7,12–17)^. A cross-sectional study using nationally representative samples detected aflatoxin B1-lysine adducts, biomarkers of chronic exposure, in 78 % of adult serum specimens, indicating widespread exposure across Kenya irrespective of age, gender and socio-economic status^(18)^. However, there is a dearth of information related to understandings and perceptions of aflatoxin among caregivers of children who may experience growth faltering as a result of exposure to aflatoxins from ingesting contaminated cereal-based foods and milk during infancy. Most studies have focussed on agricultural practices in eastern Kenya^(19,20)^, and few studies have comprehensively investigated post-harvest cereal management practices and their drivers.

A mixed methods study was conducted in western Kenya, where stunting affects close to a fourth of children 6–59 months of age and stunting is high according to international thresholds^(21,22)^ and where few data exist on aflatoxin exposure and post-harvest processing and grain conservation practices. The objective was to determine local practices related to crop management and their drivers among farmer caregivers of children under 5 years of age and to identify modifiable factors to reduce aflatoxin exposure in these households. The study was carried out in a context where few data exist on knowledge of and attitudes towards aflatoxin among mothers and caregivers of children practicing subsistence farming.

## Methods

### Study site and setting

Asembo is a rural community of largely mixed subsistence farmers in Siaya County western Kenya, with two rainy seasons annually, including the long rains (March–August) and the short rains (October–December). The population, which is mainly from the Luo ethnic group, relies on mixed subsistence farming as the main source of livelihood. Literacy rates in Siaya County are 80 %, and poverty is high, with the agricultural sector providing approximately 61 % of all employment opportunities^(23)^.

### Overview of study

A mixed methods study was conducted from August 2018 to November 2019. The quantitative study was part of a larger study investigating the effects of aflatoxin exposure on growth among children in a cohort of families, while the qualitative component included mothers or caregivers from the quantitative cohort as well as key informants with expertise in public health and agriculture.

### Quantitative methods

#### Study design and participant recruitment

The main study used a prospective design comprising serial cross-sectional interviews and food and human blood sample collection during four household visits spaced 3 months apart from August 2018 to November 2019. To facilitate enrolment, we held a public gathering with community leaders and residents to introduce the study, and subsequently, these community leaders disseminated information about the study during village meetings and household visits. The main study aimed to select at least 250 households based on sample size calculations. The sampling inclusion criteria included households with at least one child ≤ 5 years and a primary caregiver ≥ 16 years of age.

#### Data collection procedures

During the first visit, a semi-structured questionnaire was administered through face-to-face interviews with the caregiver. We collected data on household socio-demographics, food sources and consumption practices, including a directed 24-hour food recall, as well as information on cultivation, harvesting and post-harvest practices, crop storage and knowledge of aflatoxins. During the subsequent three visits, we did not assess knowledge of aflatoxin, but information on food sources, consumption, processing and storage practices was collected.

#### Data analysis

STATA statistical software version 15·1 (StataCorp LLC 2017, Release 15) was used to analyse information on household socio-demographics, planting, harvesting and post-harvest practices and knowledge of aflatoxin. Means or medians for continuous variables and proportions were computed for categorical variables. The household wealth index was generated from multiple socio-economic variables using multiple correspondence analysis technique^(24)^. Household assets and characteristics such as livestock ownership, fuel source, household material, water source and average monthly income were included in the multiple correspondence analysis with continuous variables converted to categorical variables while generating the factor effects from these sets of variables. The household wealth index was used as a proxy for socio-economic status and was classified into five wealth quintiles based on the wealth index value (1 = lowest, 2 = second lowest, 3 = middle, 4 = fourth, 5 = highest).

#### Qualitative methods

From July to August 2019, we carried out focus group discussions (FGD) with caregivers enrolled in the main study and key informant interviews (KII).

#### Study design, sampling and methods of measurement

FGD: All caregivers enrolled in the main study were eligible to participate in FGD. We established an initial target to carry out four FGD with younger caregivers between 18 and 34 years of age and four FGD with older caregivers ≥ 35 years of age and to include 8–12 participants in each group. Using the list of participants enrolled in the main study, we created two lists of caregivers according to whether they were in the younger or older age category. Following the order by which enrolled participants appeared on the lists, we purposively identified potential respondents, with the goal to ensure a relatively even distribution of participants from each age group across the ten villages included in the cross-sectional study. In preparation for each FGD, we reached out to twelve eligible respondents through verbal solicitation and phone calls to invite them to participate. Main topics explored during FGD included crop planting, harvesting and post-harvest practices, household food sources, food processing and storage, food preparation and perceptions of food spoilage. Participants were questioned on each discussion topic until no new information emerged.

KII: Eligible key informants included community members with expertise in agricultural practices and food consumption and officials from the ministries of health and agriculture working in cereal production, food safety and nutrition with first-hand knowledge of the study communities. Initial key informants were selected purposively based on their background and expertise, and after completion of these interviews, snowball sampling was employed to identify other informants.

We contacted eligible KII participants through phone and written invitations and, if they agreed to participate, subsequently approached them for enrolment. Topics explored included community food sources, residents’ behaviours related to food consumption, processing and storage, and local perceptions of food spoilage. With health and agriculture experts, we also examined community knowledge of aflatoxin contamination and decontamination.

#### Data collection procedures

Qualitative data was collected by a team of two male and two female experienced qualitative research assistants, all with educational backgrounds in the social sciences and a demonstrated proficiency in speaking, reading and writing in Luo, the predominant language spoken in the research area. Supervision of data collection was provided by a senior qualitative researcher.

The team was trained on the study protocol, and during the training, research assistants piloted and adapted the data collection tools, which were translated and back-translated into Luo. Researchers administered FGD in Luo and held discussions in community meeting halls where privacy could be maintained, with sessions limited to 2 hours. Following a guide reflecting the original study themes, a moderator led the discussions. Another researcher took handwritten notes to facilitate data transcription.

The research team administered KII in English or Luo, depending on the preference of the informant. Research assistants held interviews in key informant’s respective places of work, with interviews limited to 45 min. Since KII were conducted after the FGD, researchers added questions to the original KII study guide based on common themes that emerged from the preliminary FGD data analysis. Qualitative data collection continued until data saturation was reached.

#### Data analysis

Research assistants first translated audio-recorded KII and FGD into English and subsequently transcribed the translations verbatim in Microsoft Word version 10 (Microsoft). The research team developed separate coding systems for FGD and KII using initial research themes and questions and key concepts that emerged during the research. A codebook with detailed definitions was developed by the researchers. To ensure methodological rigour, coding of KII transcripts was done by two investigators working separately on ATLAS.ti version 8 (ATLAS.ti Scientific Software Development GmbH), a text-organizing software. An inter-rater reliability test was initially performed to compare and align coding. Content analysis was used to identify trends of concepts in and across individual codes. Throughout the analysis, validation was achieved through an iterative process of discussion and revision between study investigators. The combination of data and methodological triangulation facilitated data analysis across research methods (KII, group discussions) and between respondents.

## Results

### Quantitative results

#### Description of respondents

The main study enrolled a total of 250 households situated in ten villages. The mean household size was 6·2 persons, and all child caregivers were female with an average age of 31 years. Over 67 % of respondents had finished primary school, and the rest had completed secondary education or above. The average household monthly income was 70·40 USD, with more households in the highest compared to the lowest wealth quintile. At the time of enrolment, 40 % of children were 24 months of age or younger, with 29 % of children breastfed (Table 1). Most caregiver–respondents were involved in farming.

**Table 1:** Socio-demographic characteristics of 250 households and caregiver-child pairs enrolled in the quantitative aflatoxin study component in Asembo, Kenya

Variable *n* 250	*n*	%
Household size		
Mean	6·2	
sd	2·0	
Caregiver age		
Mean	30·9	
sd	8·2	
Caregiver education		
Primary level	167	67
Secondary level and above	83	33
Average household monthly income ($)	70·4	
Households by wealth quintiles		
Lowest (–1·16 – –0·50)	31	12
Second (–0·49 – –0·18)	49	20
Middle (–0·17–0·15)	56	22
Fourth (0·17–0·47)	51	20
Highest (0·48–1·18)	63	25
Age of children at enrollment		
0–24 months	100	40
25–59 months	150	60
Breastfeeding children	73	29

#### Crop production, processing and storage practices

Respondents reported that their households most commonly grew maize (84 %), followed by sorghum (66 %) and groundnuts (28 %), while cassava and millet were grown by few households (8 %). Data showed an increase in maize cultivation in 2018, which corresponded with a decrease in sorghum, groundnut, millet and cassava cultivation.

Most households (87 %) reported sorting harvested crops by removing visibly spoiled grains after harvesting. More households sun-dried unshelled maize (25 %) on the ground as compared to shelled maize (1 %). A majority of households used polypropylene sacks for the storage of cereals, while a small number (4 % of households) reported mixing old and new grain during storage (Table A1).

#### Crop availability and daily consumption practices

Over the course of four visits carried out in 1 year, maize was most frequently available (63 %) followed by sorghum (50 %), groundnuts (20 %) and cassava (13 %). Millet was the least available crop during all four visits (Table 2). The majority of households consumed flour made of composite mixtures of two (91 %) or more crops (96 %), with maize flour most commonly mixed with sorghum flour (58 %) or millet flour (28 %) (Table A2).

**Table 2:** Availability of cereal types, legumes and root crops in households in Asembo, western Kenya between 2018–2019

Visit number*	Maize		Sorghum		Millet		Groundnuts		Cassava	
	*n*	%	*n*	%	*n*	%	*n*	%	*n*	%
Visit 1 (*n* 250)	223	89	181	72	13	5	74	30	34	14
Visit 2 (*n* 233)	161	69	125	54	13	6	45	19	25	11
Visit 3 (*n* 230)	94	41	85	37	10	4	32	14	36	16
Visit 4 (*n* 225)	116	52	82	36	10	4	39	17	28	12
Overall	594	63	473	50	46	5	190	20	123	13

* Visit 1 – August to November 2018, Visit 2 – December to February 2019, Visit 3 – March to June 2019, Visit 4 – June to September 2019.

We asked caregivers whether children had consumed cereal-based foods in the 24 h preceding the survey. Throughout the 12-month study duration, most children consumed cereals multiple times daily either in the form of porridge or *ugali* (a stiff porridge predominantly made of maize flour and eaten with cooked vegetables and stew). Specifically, porridge was consumed by 44 % of the children while *ugali* was consumed by 95 % of the children in the last 24 h across the four visits. The results highlight age differences, with children ≤ 24 months consuming porridge an average of 2·8 times daily, compared to *ugali* which was consumed 1·7 times daily. In contrast, children ≥ 25 months consumed porridge and ugali equally (1·6 times *v*. 1·9 times, respectively) in the last 24 h (Table A3).

#### Perceptions of crop spoilage and knowledge of aflatoxin

Caregivers reported insect infestation (66 % of respondents), the presence of rot (51 %) or mould (43 %) and dampness (34 %) as the most common ways to identify spoilage. Caregivers cited inadequate drying (69 %) and poor storage (64 %) as the main causes of crop spoilage.

Overall, 51 of 250 (20 %) caregivers had heard of aflatoxin. Of these caregivers, 32 of 51 (63 %) had received information on aflatoxins from formal sources, including mass media, government officers, health workers or schools. A large majority of the fifty-one caregivers who had heard of aflatoxins (43, 84 %) expressed concern that they could become sick from aflatoxins. Caregivers reported maize (38/39, 97 %) and sorghum (21/39, 54 %) to be the most likely foods contaminated by aflatoxins (Table 3).

**Table 3:** Perceptions of crop spoilage and knowledge of aflatoxins among caregivers of children aged below 5 years in Asembo, Kenya

	*n*	%
**Perception of crop spoilage *n* 248**		
Identification of spoilage		
Insect Infestation	163	66
Rot	127	51
Mold	106	43
Dampness	84	34
Discoloration	46	19
Rotten smell	46	19
Sour taste	40	16
Perceived causes of spoilage		
Inadequate drying	173	69
Inappropriate storage	159	64
Rotting	35	14
Weevils/pest infestation	17	7
Harvested before adequately dried	15	6
Not treated with pesticides before storage	13	5
Other causes	12	5
**Knowledge of aflatoxins *n* 250**		
Heard of aflatoxins*		
Yes	51	20
No	199	80
Main source of information on aflatoxins (*n* 51)		
Formal sources†	32	63
Informal sources‡	19	37
Worry could become sick from aflatoxins (*n* 51)		
Yes	43	84
No	8	16
Knowledge that aflatoxins cause cancer (*n* 50)		
Yes	24	48
No	25	50
Do not know	1	2
Foods believed to expose people to aflatoxins (*n* 39)		
Maize	38	97
Sorghum	21	54
Groundnuts	9	23
Cassava	5	13
Milk	3	8
Millet	3	8
Other	10	26

* Aflatoxins were described in the local language to ensure respondents understood the question.

† Formal sources of information included mass media, government officers, health workers, research officers and schools.

‡ Informal sources of information included neighbours, relatives and village channels such as shopkeepers and farmers.

### Qualitative results

#### Description of qualitative respondents

Seven FGD comprising of 8–12 female caregivers were held. Because we rapidly reached data saturation with groups of caregivers ≥ 35 years of age, and there were no major differences in responses between the different age groups of caregivers, we stopped holding discussions after completing three discussions with this age group. Most FGD participants practiced subsistence farming.

Key informants included eight Siaya County health and agriculture representatives. Health and nutrition experts comprised a disease surveillance officer, two nutritionists, a maternal and child health researcher, one public health nurse involved in aflatoxin prevention and a community health supervisor working on policy development and implementation. Agricultural experts comprised an extension agent working with farmers to improve agricultural practices and an officer overseeing development programmes including those related to aflatoxin prevention. Additional key informants constituted five community residents engaged in cereal production and conservation and included one cereal vendor, an agrobusiness owner and a mill operator, as well as two experienced mother farmers with knowledge related to traditional and current agricultural practices. A summary of the FGD and KII participants is given in Table A4.

#### Cultivation, harvesting and post-harvest practices

Key informants stated that most Asembo residents practiced subsistence farming, attributing a preference for maize cultivation to good yields and versatility in food preparation. All types of respondents reported recent alterations in the timing of planting and harvesting due to changing weather patterns involving late and erratic rainfall and higher moisture levels, which they claimed to be causing reduced crop yields. Focus group participants asserted that persistent reductions in crop yields affected household food stocks and pushed farmers to harvest crops prematurely before they were fully dry. Harvesting crops early was also mentioned as a strategy used to avoid crop theft. One group participant said

*In the past, people harvested crops when they were ready, but now hunger is making us harvest when crops are still not well dried. This is causing us problems. Sometimes we harvest crops from the farm early because we are hungry. Sometimes we harvest early because people steal from the farm. We feel that we need to harvest all of the crops and store them, despite the fact that they are not ready… This also leads to pest infestation.* (Focus group participant in the 18–34-year-old caregiver group)

Key informants and focus group participants reported that post-harvest rains and cloud cover, as well as higher precipitation during the rainy season, increased challenges to drying crops. A community key informant said

*When the crops are ready for harvesting, the rains continue and sunlight is scarce. After the harvest, there are no dry areas to place the cereal outside and farmers are forced to put the grains in the house where space is limited. And when they bring the crops outside for drying, then the rains start again. So, the house is stuffed with cereal until farmers discover that the cereals have turned into stones, they become green, due to the weather they cannot be dried well, and the grains become rotten.* (Key informant, Mill operator)

Respondents stated that farmers sun- and air-dried crops. Group participants reported sun-drying crops directly on the ground, with some specifying that this is a common practice with unshelled maize and groundnuts, and that shelled maize can also be dried on the bare ground. Group participants did not associate drying crops on the bare ground with spoilage, whereas key informant agricultural officials claimed that drying shelled groundnuts and cereals directly on the ground is a common practice and a major cause of cereal spoilage.

Government agriculture officials asserted that local farmers commonly combine unspoiled and spoiled cereals, as well as old and new harvests due to a reluctance to discard food. One nutritionist reported the belief that mixing good and spoiled or damaged cereal dilutes crop spoilage. Focus group participants and community key informants mentioned that farmers add ash to prevent pests such as weevils and worms from attacking stored cereals. Some group participants also reported applying pesticides to stored grains, although they considered pesticides less effective than ash and unaffordable to some residents.

Key informants and group participants stated that granaries are no longer used because of concerns related to theft. Community key informants maintained that granaries are less essential due to reduced crop yields, adding that the skills to build granaries have been lost over time. Key informants and group participants reported that families store farm-grown crops on the ground inside the family house. The exception is groundnuts, with focus group participants indicating that families hang groundnuts from the roof to avoid exposure to moisture and protect from pests, especially rats, which are considered a major source of groundnut spoilage. Group participants mentioned that household size prevents most families from designating a specific storage area for harvested grains and that families often store cereals in rooms where household members or animals sleep, with participants indicating that animals sometimes get access to and scatter stored crops on the ground. Focus group participants acknowledged that placing storage containers directly on the ground increases exposure to moisture, while key informants claimed this to be a common practice, causing cereal spoilage. When describing the challenges of storage, one group participant stated

*Sometimes the house is small, it is just one room, children sleep here, adults also sleep here, and the cereals are also here. Farmers may not even have money to buy a sack, so they put cereals on the floor, and the cereals get stepped on and scattered* (Focus group participant in the ≥ 35-year-old caregiver group).

Most focus group participants reported storing cereals in polypropylene sacks followed by jerricans and barrels, which participants explained cannot be infiltrated by pests such as rats and weevils. While they mentioned that community members know about hermetic bags, group participants stated that hermetic bags cost 1·50 USD and are generally considered unaffordable compared to polypropylene sacks sold for 0·50 USD.

#### Food acquisition and consumption

Due to limited land availability and harvest outputs, focus group participants and key informants noted that most residents are unable to rely solely on homegrown crops for household food consumption. Qualitative data elucidated that subsistence farming households first consume homegrown cereals, and once depleted, purchase cereals from the market which are sourced from neighbouring counties. The exception is millet, which is rarely grown in the region. Focus group participants reported that maize constitutes the main ingredient for *ugali* and porridge, with children’s porridge composed of maize, sorghum, millet and groundnut flour.

Group participants expressed concerns about purchased grains, stating they cannot know how market cereals are processed post-harvest and underscoring greater confidence in the management of their own crops. Some mentioned that market cereals are sometimes discoloured or mouldy. Many claimed that consumption of purchased cereals causes abdominal pains and diarrhoea, unlike homegrown cereals. A focus group participant said

*Grains that we purchase give us (health) problems because they are not well prepared… These grains are just taken from the farm and shelled with all the chaff, they are shelled and put into a sack, they are never dried, and so they are brought to the market spoiled. We know that the grains from our farms are dried and shelled and winnowed before they are mixed with ash and stored.* (Focus group participant in ≥ 35-year-old caregiver group).

When describing the potential risks of eating cereals sold in markets, another participant added

*I bought cereal from the market, and after I cooked it, everyone in the house had diarrhea. I don’t like buying cereals, they have problems, they cause stomach problems to family members.* (Focus group participant in ≥ 35-year-old caregiver group)

Government officials noted that cereals sold in markets are transported in open trucks and frequently exposed to rainfall, and because traders want to sell products quickly, the cereal is not aired properly, with traders concealing or removing grains showing signs of spoilage.

#### Perceptions of crop spoilage

Focus group participants reported mould, holes, discolouration and a sour taste as signs of spoilage, with community key informants mentioning that spoiled grains become sticky. Some group participants suggested that spoiled grains are used as animal feed or to brew alcohol. One group participant said

*After you harvest grains from the farm then you separate the rotten ones from the good ones so that they are not mixed together. The rotten ones are given to chicken or used to brew alcohol.* (Focus group participant in ≥ 35-year-old caregiver group)

Focus group participants mentioned weevils as the insect most frequently causing cereal spoilage, both before and after harvest. They reported recent increases in pest infestation, which farmer participants associated with reduced yields and food stocks. Community key informants stated that local farmers weed in an effort to reduce pest infestation and improve crop yields.

#### Local understandings of aflatoxins

Key informants claimed that community members have little to no knowledge about aflatoxins and the corresponding health consequences such as cancer. It was noted that crop management practices are primarily guided by efforts to enhance crop yields and the foods available for household consumption. One government official said

*That (aflatoxin) is not even in their mind. They try to control rats, and other things, like weevils… That is what is most important.*

Most key informant county representatives acknowledged little or no information sharing on aflatoxins, emphasising the need for better outreach to sensitise residents about food safety and to control food quality. They recommended increasing extension workers to assist farmers with improved crop management practices.

## Discussion

Our findings elevate concerns surrounding risks of foodborne exposure to aflatoxins in young children in Asembo and the associated health effects, including growth faltering, which is reported to affect close to a fourth of children between 6 and 59 months in Siaya County^(25)^. Children living in farming households consumed cereal-based staples multiple times daily starting from a young age. Mothers and caregivers had limited knowledge of aflatoxin.

High intake of cereal-based food by children in this study population is noteworthy. Other studies in sub-Saharan Africa have found that dietary exposure to aflatoxins increased after weaning and linked growth faltering and underweight in young children to high aflatoxin levels in cereals^(26–28)^. Moreover, children exposed to aflatoxins early in life are likely to suffer disproportionately from the long-term effects of aflatoxin contamination compared to adults^(29,30)^. Results showed that higher crop yields influenced subsistence farmers to primarily cultivate maize and sorghum and mix flours derived from these crops to make *ugali* and porridge. Frequency of consumption of maize-based products has been identified as a potential risk to aflatoxin exposure and a likely explanation for periodic aflatoxicosis outbreaks in Kenya causing acute illnesses and death^(31,32)^. Research also shows that aflatoxins are higher in composite flours^(33)^.

Results revealed that subsistence farmers applied several crop management and conservation practices shown to reduce fungal activity. However, economic constraints and changing environmental patterns created barriers to other post-harvest handling, processing and storage practices known to lower moisture levels in crops, thus raising risks of exposure to aflatoxin contamination. Climate shifts suggest the problem may get worse in a setting where farmers struggle to maintain adequate crop yields for subsistence purposes. In a country context where previous serosurvey found that 78 % of adults had detectable levels of aflatoxin, only 20 % of smallholder farmers were aware of aflatoxins, with far fewer familiar with the harmful health consequences. Findings suggest that from a young age children living in smallholder farming households may be at risk of exposure to aflatoxin contamination and its health implications^(5,6,29,30,34)^.

Our study results highlighted that because farm yields can be insufficient to sustain household food consumption into the next harvest, farmers are forced to purchase cereal from the market after home-produced cereals are depleted. Study participants reported signs of spoilage and negative health effects following the consumption of market foods, raising concerns about the management of market-sold foods. A study conducted in a neighbouring county in western Kenya found that market-purchased sorghum had higher levels of aflatoxin contamination as compared to homegrown cereals, validating concerns raised by our study respondents^(35)^. Other research in Kenya and elsewhere have identified a wide range of food commodities contaminated with high levels of aflatoxins, particularly maize and peanuts^(4,12,13,36,37)^.

Study respondents linked signs of crop spoilage to pest infestation and dampness caused by inadequate drying and poor storage, all conditions which lead to aflatoxin contamination^(4)^. Findings highlighted the application of post-harvest measures proven to prevent spoilage and thus decrease aflatoxin contamination, such as the removal of visibly spoiled cereals, sun-drying cereals on canvas, mixing ash with stored cereals and storing cereals in containers. However, economic factors guided the application of a multitude of other sub-optimal practices including early harvest of crops, poor crop drying and storage techniques and mixing grains exhibiting signs of spoilage with good cereals to minimise loss of cereal destined for household food consumption. The phasing out of traditional granaries and storing of cereals in polypropylene bags and confined quarters occupied by humans and livestock also reflected poor economic conditions. A study conducted in eastern Kenya found that the densities of aflatoxin producers are 71 % higher in cereals stored in polypropylene bags compared to those stored in hermetic bags^(19)^. Other research has shown that indoor storage and placement of cereals directly on the ground decreases ventilation and increases condensation and humidity, providing conditions for fungal growth^(38,39)^. Storing grains in locations shared by humans and animals increases the likelihood of introducing foreign materials, less hygienic conditions and damaged grain, which can lead to fungal invasion and the production of aflatoxins^(40–42)^. Use of spoiled cereals for animal feed and the production of traditional alcohol, which in this context was commonly made of maize, may also increase exposure to aflatoxins, as has been demonstrated in studies in Kenya and elsewhere^(43–45)^.

Qualitative study participants consistently described changing and unpredictable rainfall patterns that appeared to impact farmers’ abilities to dry cereals and can lead to increased moisture levels in stored cereals and pest infiltration. Smallholder farmers in our study, whose average monthly earnings placed them below the extreme poverty line^(46)^, had limited means to make adaptations to protect against the negative effects of climatic fluctuations. Other studies have shown that subsistence farmers who rely on rain-fed agricultural practices are particularly vulnerable to climate change^(7)^, which in Kenya is manifested by variations in seasonal precipitation and increased and variable temperatures^(47,48)^. A recent assessment carried out in Siaya County projected that increased weather fluctuations would constrain future agricultural productivity and compound challenges to crop conservation^(23)^. These environmental changes, which are occurring in a context where inadequate drying and humid storage conditions already exist, will likely compound conditions for aflatoxin contamination.

Without adequate control and prevention measures, residents of western Kenya and their children will continue to be at risk of exposure and the associated economic and health consequences of aflatoxin contamination. Strategies recommended to decrease exposure in settings where aflatoxins are prevalent involve prevention and control of fungal growth in crops, decontamination of aflatoxin-contaminated foods, continuous surveillance of aflatoxins in human and animal food crops, application of positive post-harvest management practices and regulations to protect consumers from the harmful effects of aflatoxins in foods^(1,4)^. While Kenya has a national food and nutrition policy to address food safety and has adopted maximum limits of aflatoxins in foods, inadequate equipment, human resources and financial support impede policy enforcement of the application of essential measures to ensure quality assurance of food commodities. Investments are needed so that key strategies related to food safety such as training on quality control, surveillance of market products and food testing are widely implemented. In addition, ongoing research to ensure the safe use of biocontrol measures to mitigate aflatoxins should be continued.

Results from our research and other studies in neighbouring Tanzania and the Democratic Republic of Congo where aflatoxins are prevalent, highlight low awareness of aflatoxins and their deleterious health consequences, underlining inadequate information sharing with smallholder crop producers^(49–51)^. Efforts are needed to better inform farmers about the risks of aflatoxins and to equip them with practical knowledge and tools to follow good agricultural practices aimed to prevent and control contamination. Approaches could promote existing post-harvest processing and storage practices shown to improve crop conservation and thus impact on livelihoods, reduce aflatoxin contamination and enhance food safety in settings where aflatoxin contamination and exposure are known to be high. Strategies may need to consider local conditions and constraints, including economic and changing environmental challenges that guide farmers’ decisions related to crop management and their ability to adapt practices.

### Study limitations

Quantitative and qualitative data collection was carried out with the same study population, which could introduce research bias by exposing the focus group participants to more information and creating higher awareness of aflatoxins among these caregivers. The relatively short study time period did not allow us to investigate how patterns in climatic change and economic fluctuation affect post-harvest practices over longer time spans. Although the study lacks information on actual exposure to aflatoxins, the research was conducted in a context where the prevalence of aflatoxins has been established.

## Conclusions

Evidence of persisting aflatoxin contamination in Kenya underlines the need for sustained efforts to increase public awareness and implementation of effective aflatoxin control measures among subsistence farmers. Research is required to determine which pre- and post-harvest practices would be feasible, acceptable and effective in preventing and controlling aflatoxins in cereals that smallholder farmers living in western Kenya rely on for livelihoods and household food consumption. Subsistence farmers may need special assistance adapting agricultural practices to recent and ongoing climatic changes that can lead to increased moisture levels in stored crops and risk of aflatoxin contamination. Social behavioural change approaches aimed to increase understanding regarding the deleterious effects of aflatoxin food contamination and to motivate improved agricultural practices should be considered. The mixed methods research approach provided special insights into the complexity of factors and practices underlying potential aflatoxin contamination.
